# Healthy Food Labels Tailored to a High-Risk, Minority Population More Effectively Promote Healthy Choices than Generic Labels

**DOI:** 10.3390/nu11102272

**Published:** 2019-09-22

**Authors:** Christopher R. Gustafson, Michael R. Prate

**Affiliations:** 1 Department of Agricultural Economics, University of Nebraska-Lincoln, Lincoln, NE 68583, USA; 2 Rosebud Food Sovereignty Initiative, REDCO, Mission, SD 57555, USA; michael.prate@sicangucorp.com

**Keywords:** behavioral economics, health disparities, food labeling, choice experiment, minority

## Abstract

The decades-long increase in obesity in the US has led to a number of policies aimed at improving diets, which are thought to play a significant role in obesity. Many of these policies seek to influence individuals’ behaviors. Front-of-package labels providing salient, easily interpretable information to consumers have exhibited promise in helping people identify and choose healthier foods. However, behavioral economics may offer an opportunity to enhance label effectiveness. Tailoring labels to high-risk communities, including minority and rural populations, which have higher rates of diet-related diseases than the overall population, may increase the label’s effectiveness. We conducted a choice experiment with supermarket shoppers on a rural American Indian reservation to test labels tailored to the local population relative to a generic label, which had previously been identified as highly effective in the general population. Results show that while the generic label continues to be quite effective in encouraging healthier choices, the label that is tailored to the local community is more effective, resulting in a marked increase in the premium shoppers were willing to pay for a healthy item. Tailoring healthy food labeling systems using insights from behavioral economics may increase their effectiveness.

## 1. Introduction

The prevalence of obesity in the United States has increased markedly over the past few decades and currently affects close to 40 percent of the adult population [1]. Obesity is linked to a variety of negative consequences for individuals and society. These consequences include poorer health, an increased risk of associated non-communicable diseases, such as type-2 diabetes, certain types of cancer, and heart disease, and reduced life expectancy [2]. Higher rates of obesity lead to increased health care costs and other negative economic impacts that are borne both privately and publicly [3,4].

Dietary quality is frequently cited as a main contributor to the rise in obesity rates. In response, the primary policy strategy used by the federal government to address obesity has been to provide nutrition information directly to consumers. The Nutrition Labeling and Education Act (NLEA) of 1990 requires food manufacturers to display nutritional information on packaged food products, and the Affordable Care and Patient Protection Act (ACA) of 2010 stipulates that calorie information be posted in restaurants with 20 or more locations [5]. However, studies of the effects of the NLEA and early adopters of restaurant calorie labeling show little effect on the nutritional quality of individuals’ food choices. Efforts to make nutritional information easier for consumers to use in the retail environment have led to the development of simple shelf-based or front-of-package labels. A recent, large scale investigation of one retail shelf-based system finds positive shifts in the overall healthiness of foods purchased, though the results are predominantly driven by a reduction in the quantity of unhealthy foods purchased, rather than by an increase in healthy foods [4].

While average obesity rates have risen significantly in the US, these averages mask important differences in obesity rates by demographic and socio-economic variables, including race, income, and place of residence [1,6]. In general, minority, rural, and poorer households experience higher rates of overweight and obesity than the general population [1,6,7]. African American and Hispanic individuals have been found to have significantly higher rates of obesity than white individuals of the same age groups [8]. American Indians are among the most at-risk groups for obesity at all ages [9,10].

In this study, we aimed to examine whether tailoring healthy food labels to a high-risk community—residents of a rural American Indian reservation in the Great Plains—would influence how effective the materials were in promoting choices of healthier foods. Labels were tailored to the community through the involvement of community members in the development of the materials. We tested the effect of the tailored label relative to a generic label that has been found to be effective in a multinational sample of representative consumers [11]. We were interested primarily in the effect of labels on community members who had not yet been involved in the development of the labels as a conservative measure of the effect of tailored labels, since research shows that involvement amplifies response to healthy food materials [12]. The tailored label used in the research was designed by a small group of local residents and researchers who collaborated on a food sovereignty project initiated by the tribal government. None of the collaborators on the project completed surveys.

While health promotion efforts aimed at minority communities are often designed to be culturally appropriate, research in behavioral economics and psychology presents reasons why tailoring materials to a community may increase the effectiveness of health promotion materials. Tailored labels that feature community involvement in the design may communicate social norms, which have been found in other contexts to influence attitudes and intentions towards foods [13,14]. Particularly among low income communities, tailored labels may invoke positive elements of identity, which may influence response to food [15]. Design efforts that engage the community more broadly in the design process may also increase effectiveness through involvement [12].

In this article, we examine the effect of healthy food labels in the choice of healthier and less healthy food items in a choice experiment conducted with people actively shopping in a supermarket on the Rosebud Indian Reservation in Mission, South Dakota. A choice experiment allows us to explicitly compare trade-offs between price and health with and without a healthy food label in place. Previous, related research had not considered differences in cost between healthier and less healthy products, which is frequently cited as a barrier to healthier eating, particularly among low income households [16,17].

## 2. Materials and Methods

We conducted the research in a supermarket in Mission, South Dakota from October 9–11, 2015. Mission is the largest incorporated community on the Rosebud Indian Reservation, which is located in south-central South Dakota. The Rosebud Indian Reservation is the home of the Rosebud Sioux Tribe—over 90% of Reservation residents are fully or partially Native American [18]. Residents of the Rosebud Indian Reservation have many of the characteristics that are associated with poorer health outcomes. Poverty is widespread. The per-capita income at the time of the study was under $12,000, and nearly 50% of residents lived in poverty [18]. Additionally, educational attainment is markedly lower than the US average, with less than 15% of adults having completed college [18]. All of these factors are associated with lower quality diets and poorer health.

We examined the effectiveness of labels informed by behavioral economics by comparing three healthy food labels: a label featuring images and text tailored to the Rosebud population, a generic label that had been found to be highly effective at helping people identify and choose healthier foods among a large international sample [11], and a control label that included only the imagery incorporated into the tailored label. We included the third label to control for the effect of the culturally relevant symbol used in the tailored label.

**Figure 1:**
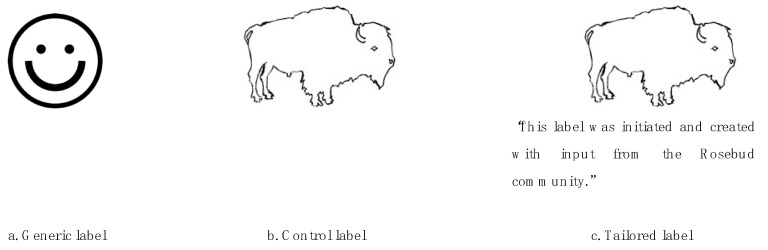
The three versions of the healthy food labels observed by choice experiment participants.

The tailored label was developed by collaborators from the Rosebud Food Sovereignty Initiative, Sinte Gleska University, which is the tribal university of the Rosebud Sioux Tribe, and the University of Nebraska–Lincoln. Primary input for the design of the label came from local collaborators. The tailored (and control) label featured an image of a bison. The bison image was identified by local collaborators as a culturally relevant image associated with notions of health and strength. Therefore, we hypothesized that the image would invoke participants’ cultural identity, rendering the label more salient and prompting participants to consider health attributes. The tailored label included text around the bison image stating that the label was the product of a local, community-led initiative, which may communicate injunctive social norms [19,20]. We hypothesized that the injunctive norm messaging would increase the likelihood that people would choose the healthier item relative to the generic label. The generic label was chosen based on previously published research that found the image—a smiley face—to be highly effective for the average consumer [11]. Label images are displayed in Figure 1.

Shoppers were recruited shortly after they entered the supermarket to participate in a choice experiment in order to examine the effect of the three labels on the healthfulness of food choices. Those who expressed willingness to participate completed a written informed consent process, and then received written introductory material and instructions that contained explanations of the choice task (these materials and an example choice set are included in Appendix A). Although shoppers read through the materials on their own, researchers were available nearby to answer any questions. These written materials also included a cheap talk script, which has been shown to reduce gaps between choices made in hypothetical and non-hypothetical decisions in a range of settings [21,22], including in choice experiments [23]. After reading through these materials, the participants completed the choice experiment, followed by a short demographic survey. The instructions and choice experiment were conducted on paper due to unreliability of internet access at the study site.

Each participant made purchase decisions in eight choice sets. Each choice set contained a healthier product, a less healthy product, and an opt-out statement (“I would not buy either of these products”). In four of the eight choice scenarios, participants saw the healthy label applied to one of two types—either unfrosted corn flakes or unfrosted shredded wheat—of healthier products. In the other four choice scenarios, no label was applied to the other type of healthier product, thereby serving as a control condition. Appendix B displays an example set of product attributes included in each of the eight choice sets for a participant who saw the healthy label applied to unfrosted corn flakes (shredded wheat serves as an unlabeled control). Note that the option to indicate “I would not purchase either of these products” is not displayed in Appendix B (but is displayed in the example choice set in Appendix A). Each participant only observed one of three the label types, and only saw that label applied to one of the two healthy product types. The other healthier product type did not receive a healthy food label. The label and labeled food type were determined for each participant by the researcher randomly drawing a paper-based survey instrument from a closed box that the surveys were transported in. In every choice scenario, the participant could select the healthier item, the less healthy item, or indicate that they would purchase neither product.

The products used in the choice experiment were bagged cereals: two corn-based cereals and two shredded wheat-based cereals. We chose to use breakfast cereals in the experiment following discussions with local project collaborators who identified breakfast cereal as a product that most local residents commonly purchase. We chose to use this product for two reasons. First, participants’ preferences for commonly consumed foods are likely to be more well defined and, therefore, decisions made in the context of the choice experiment should be more stable than for products that are novel or infrequently purchased, providing a more meaningful test of the effect of the healthy food labels. Second, and perhaps more importantly, changes in decision-making about foods that are consumed regularly will have more of an impact on overall nutrient consumption and dietary quality than changes in foods that are infrequently consumed.

Participants faced choice sets containing all combinations of the other attributes of interest for the corn-based and shredded wheat-based cereals. We examined participants’ choices between 40-ounce bags of healthier and less healthy cereals at two different price levels—$4.99 and $5.99—which reflect the typical range of regular and on-sale prices at the study location. The two healthier cereal varieties were corn flakes and shredded wheat, while the two less healthy varieties were frosted (corn) flakes and frosted shredded wheat.

We used mixed effects logistic regression to analyze participants’ choices. We present the results of two mixed effects logistic models with individual-specific random effects. In each regression, we examined the effect of the type of label on product choice while controlling for attributes of the product, the effect of price, and, in the second model, characteristics of the individual participants.

The first analysis contains only the product-specific attributes—price, cereal type, whether the cereal was frosted or not—and the presence and type of healthy food label. The second analysis adds demographic variables, including household size, gender, age of the respondent, household income, whether the respondent is the primary shopper for their household, and years of education. We then used parameter estimates for price and health attributes in the different labeling conditions (including no label) to calculate willingness to pay for the healthy and unhealthy attributes in the presence of the three different label types, and when no label is present. These estimates were used to identify how labeling in general and the use of the tailored (vs. generic) labels influence valuation for health attributes.

All subjects provided gave their informed consent for inclusion before participation in the study. The study was conducted in accordance with the Declaration of Helsinki. The protocol was approved by the University of Nebraska–Lincoln’s Institutional Review Board (#20150815457 EX) and Rosebud Sioux tribal authorities. We consider *p*-values < 0.05 to be statistically significant. Data were analyzed using R (R Core Team (2018). R: A language and environment for statistical computing. R Foundation for Statistical Computing, Vienna, Austria. URL https://www.R-project.org/) [24].

## 3. Results

We received 115 complete surveys. We received 35 surveys in the tailored label condition, 31 surveys in the control label condition, and 49 surveys in the generic label condition. Summary statistics on participants are presented in Table 1. Ninety percent of participants reported being the primary shopper for their household, and 71 percent of participants were female. The mean age of participants was just under 42 years. Participants reported completing approximately 13.5 years of education, with 91 percent of participants having achieved at least a high school diploma. Household income was reported to be quite low. The average annual household income for the sample was approximately $17,000. These data generally reflect available census data on Mission, SD, the community in which the supermarket is located [18], though our participants had a slightly higher high school graduation rate (91 percent versus 82 percent), and household income is slightly lower ($17,000 for our sample versus $25,000). The latter difference may reflect the fact that there are few food retail locations on the reservation outside of this community and many residents of the more rural parts of the reservation come to this community to shop for food. With fewer employment opportunities in the rural parts of the reservation, incomes are also likely to be lower.

The regression results are presented in Table 2 (we present the proportions of choices in each labeling condition—including when no label was present—in Appendix C). The estimated parameter on the variable Price is negative, statistically significant (*p* < 0.001), and nearly identical in both regressions, with a point estimate of −0.63. Estimated label parameters are relative to the unhealthy item in the “no label” condition. Participants were more likely to select the healthy product than the unhealthy product when no label was present in the choice set (*p* = 0.02). Both the tailored and generic labels increased the probability that participants chose the healthy item (*p* < 0.001). The presence of the tailored image in the choice set also decreased the likelihood that participants chose the unhealthy item (*p* < 0.001). We also controlled for the ingredient attribute *Wheat* (relative to corn), which is positive and significant (*p* < 0.05). Of the demographic variables, only Male and Primary Shopper are statistically significant. Both increase the probability that one of the two products were selected.

We first examined how label conditions influence the probability that participants choose the healthy product. In the unlabeled condition, participants were more likely to select the healthy item at the five percent level (relative to the unhealthy product in the unlabeled condition). Both the generic and tailored healthy food labels increase the probability that participants chose the healthy item, and both were statistically significant (*p* < 0.001). Interestingly, the control label was not statistically significant, though the point estimate was positive. We discuss this finding at greater length later.

Next, we examined how label condition influences participants’ choice of the unhealthy product. Neither the presence of the generic label nor the control label in the choice set impacted the probability that the unhealthy item was chosen at a statistically significant level. The presence of the tailored label, however, significantly decreased the likelihood that the unhealthy item was chosen (*p* < 0.001).

The parameter estimates from the choice experiment can be used to calculate estimated differences in willingness to pay (WTP) for product attributes. WTP values were calculated by taking the ratio of the parameter estimate for the healthy attribute to the parameter estimate for price, multiplied by negative one. For instance, the willingness to pay in labeling condition m for health attribute n is calculated as

(1) WTPm,n=−βm,nβprice.

The calculated WTP values and standard errors are presented in Table 3. The standard errors used to construct the 95 percent confidence intervals were generated using the delta method.

WTP for the healthy product is $0.50 greater than the unhealthy product in the unlabeled condition and is significant at the one percent level. WTP for the healthy item in the control label condition is $0.30 and is not statistically significant. The tailored label and generic label both increase WTP for the healthy food item relative a choice set in which no label is present. When the healthy item carries the tailored label, WTP for the healthy item is $1.75, increasing WTP by over $1 relative to the no label condition, and is significant at *p* < 0.001. WTP for the healthy item when carrying the generic label is $1.48 and is also significant at *p* < 0.001.

The presence of labels in a choice set generally decreases WTP for the unhealthy items. WTP for the unhealthy item in the presence of the control label ranges from $0.05 to -$0.001, depending on whether controls for demographic variables are included in the regression. Neither of these WTP values is statistically significant. For choice sets in which the generic label is present, WTP for the unhealthy item decreases by $0.38 relative to the no label condition, though this is not statistically significant. WTP for the unhealthy item when the tailored label is present, however, is $1.20 lower than when no label is present. This estimate is significant at *p* = 0.01.

While the generic and tailored labels both significantly increase the probability that the healthy item was chosen, only the tailored label additionally decreases the likelihood that the participant selected the unhealthy item. Taken together, the difference in WTP for the healthy and unhealthy items is markedly different across labeling conditions. The difference in WTP between the healthy and unhealthy products in the no label condition is $0.50. When the generic label is present, the difference in WTP is $1.86, or $1.36 more than when no label is present. The tailored label increases the difference in WTP further. When the tailored label is present in a choice set, the difference in WTP between the healthy product is $2.94, or $2.44 more than when no label is present.

We find support for our hypothesis that simple healthy food labels that are tailored to high-risk communities may increase the effectiveness of the labels in promoting healthy food choices. Both tailored and generic labels were effective at increasing healthier choices and the tailored label was additionally effective in decreasing unhealthy choices in a choice experiment conducted with people actively shopping in a supermarket.

The findings from the choice sets with the control label, which featured the same imagery as the tailored label, suggest that the results of the tailored label may provide a conservative estimate of the potential of tailored healthy food labels to positively influence food choice. Because the imagery on the control and tailored label was identical, the difference in results must stem from the message indicating the local origin of the label in the tailored label. Without the message, the bison image itself fared poorly against the generic label image, suggesting that both imagery and message play an important role. The bison image used in this research did not undergo any testing prior to the implementation of the survey to try to “optimize” the imagery. Therefore, it may be that the tailored message, which may evoke social norms surrounding healthy food consumption, combined with a more effective image, could lead to additional gains in healthy food choice. An alternative explanation is that participants in the tailored label condition may have received sufficient context via the text accompanying the bison image to contextualize the image, while the participants seeing the bison image with no additional explanation may have not responded to the image in the way we expected—for instance, it may have led participants to think about high levels of unhealthy food consumption, obesity, and diet-related diseases that have been documented in the study area, rather than health. The image bringing to mind these thoughts could have resulted in the identity label being ineffective.

## 4. Discussion

Food choice is complex and is influenced by cognitive and biological systems that affect how we respond to rewarding stimuli, such as food, and how preferences, habits, and other considerations factor into decision-making [25]. In this research, we examined the effect of tailoring labels to high-risk communities through community involvement in the development of labels by comparing three healthy food labels in a choice experiment conducted in a rural, low-income, minority community. The labels were a generic label found to be highly effective at helping people identify and choose healthier foods among a large international sample [11], and two different labels that were targeted to the community—one of which featured text describing its local origin and imagery that had been identified as a symbol of health and one which featured only the image (without any text implying local involvement in the label design). The choice experiment also permitted explicit comparison of trade-offs between price and health with and without a healthy food label in place. The price of healthy foods is frequently cited as a substantive barrier to healthy food choice for low income households, so it is important to consider trade-offs in price in the design of healthy food promotional efforts.

Our results suggest that local involvement in the development of healthy food labeling systems can increase the purchase of healthy foods, even when prices for healthier and less healthy items vary. Since many ethnic minority and rural populations experience diet-related health problems at a rate higher than the US average, tailoring labels and other health promotion efforts—for instance, healthy food promotional materials, or public health campaigns—to the population may lead to a greater effect than generic materials.

One potential explanation for the additional effectiveness of the tailored label—but not the control label featuring the same imagery as the tailored label—relative to the generic label is social norms. Social norms highlight what an individual’s peers have chosen, referred to as descriptive norms, or communicating the choices that others believe are “good”, known as injunctive norms. The presentation of the tailored label implies an injunctive social norm valuing eating a healthy diet by stating that the label represented a local effort to promote healthy foods.

Social norms have been studied in the context of food choice in both survey-based and experimental studies [e.g., 13,14,26]. In general, findings from this research support a role for social norms in bolstering intentions to make healthy food choices [13,26,27] and in influencing actual food choices [20,28,29]. Importantly, however, none of these studies examines the effect of social norms on choices when the choice options are offered at different prices.

In existing research on the effects of social norms on food choice, price differences were either not considered in the study design (as in Burger et al., [29]), or the research was designed specifically to avoid introducing price differences [28]. We explicitly included price variation of the items in the design of our choice experiment to examine the effect of varying prices on choice. Since the cost of healthy food is frequently cited as a barrier to healthy eating, particularly for low income households [30,31], it is important to examine choices when relative prices differ.

While there is a small, but promising amount of lab and field-based literature suggesting that simple materials—for instance, shelf labels or front-of-pack (FOP) panels—that prompt people to consider health when making food choices may lead to a healthier mix of products purchased [32,33,34,35], none of these studies has tested a design tailored to high-risk populations against generic materials. Both task-based (i.e., research in which participants are instructed to identify the healthier food) and hypothetical, preference-based (examining stated preferences in the presence/absence of labels) studies have been conducted on healthy food labels in laboratory settings [11,36]. However, evidence from the field on the effects of shelf or product-based labels on choice is rare [34] or, as labeling is frequently implemented as one component of a multifaceted intervention, difficult to tease out [37,38].

Our research on healthy food labeling was conducted on an American Indian Reservation in rural South Dakota. Similar to other American Indians and Alaska Natives (AIAN), residents of the study area have high rates of obesity and diet-related diseases [10]. Over 20 percent of two to four-year-olds in the study area were found to be obese [39], which is more than twice the national average for children in this age range [40]. While current data on adult obesity rates are not available for the study area, adult obesity rates for AIAN individuals nationally are nearly 10 percentage points higher than for whites [41]. Nation-wide studies of behaviors and health outcomes of AIAN individuals find lower healthy food consumption and higher rates of obesity and diet-related disease and mortality compared to whites living in the same areas [10,42]. Additionally, life expectancy in the study area is markedly lower than the national average for both males and females [43]. Nationally, the life expectancy of AIAN populations is approximately 4.5 years shorter than the general US population [44]. AIAN individuals also report being in fair or poor health at a higher rate than other racial/ethnic groups [41,45]. Given the challenges facing high-risk minority populations, identifying strategies that can increase the effectiveness of health promotion efforts is important. Our research suggests that tailoring materials to high-risk communities in a way that communicates social norms is a potential means by which to more effectively promote healthy behaviors.

While these initial results show promise, there are some potential weaknesses of our study. The experiment involved hypothetical choices, and it is reasonable to expect that choices—and choices about healthier and less healthy foods in particular—might be different if decisions were binding. However, we implemented multiple design elements to reduce concerns about hypothetical biases. First, we used a cheap-talk script [21], which has been shown to reduce hypothetical biases across multiple data collection methods, including choice experiments [23].

Second, we were primarily interested in the changes in choices among labeling conditions (and compared to the no label control condition) rather than the absolute number of healthy choices. As Charness et al. [46] recommend, exposure of participants to label type (i.e., tailored, control, and generic) occurred as it would in a natural choice environment—with only one label type viewed by each participant. Our use of a between-subjects design for label exposure eliminates participants’ ability to consciously compare among labels. Given that two of the three labels were targeted to the local community, exposure to all of the labels would likely have led participants to evaluate each label relative to the other labels, which may have resulted in choices in the experiment being made according to a different set of criteria than the participant would have used if exposed to a single label.

Future work in this area needs to address weaknesses in this study by evaluating non-hypothetical choices. A good test of the concept would be to compare the effectiveness of generic and tailored, social norms-based labels of healthy food labels in a retail environment. Future work could also look to integrate norms that provide target levels of healthy food consumption based on the purchasing habits of consumers who purchase above average amounts of healthy foods (as has been done with energy use—see Allcott [47] or Asensio and Delmas [48]) along with injunctive norms supportive of healthy diets.

Future work could also investigate potential additional benefits from involving the community in the development of healthy food labels (rather than developing the labels without significant community input—only local members of our team were involved in label design, as was true of the labels used in this research). Research in other fields suggests that being involved in a process can boost intrinsic motivation and commitment to follow through on objectives [49] and has been found to make a difference in an experimental plate-waste study on vegetable choice and consumption with children [12]. Community involvement in the design of labels may also help establish and strengthen social norms related to healthy eating. Involving the community would also help guarantee that label design and messaging is effective and well aligned with community values prior to implementation.

## Figures and Tables

**Table 1:** Summary statistics of participants in the choice experiment.

Variable	Definition	Mean	Std. Dev.
Household Size	Number of individuals living in the household.	4.73	2.52
Female	= 1 if the respondent is female, 0 otherwise.	0.71	
Age	Age of the respondent in years	41.8	14.3
Primary Shopper	= 1 if the respondent is the primary shopper in the household; 0 otherwise.	0.90	
Education	Years of education	13.46	1.99
HH Income	Household income (measured in $1000s)	16.91	13.89

Data are from the choice experiment and demographic survey. *n* = 115.

**Table 2:** Mixed effects regression results of the influence of labels on product choice.

	Model 1: Choice Data Only		Model 2: Choice Data and Demographic Variables	
Variable	Estimate (SE)	*p*-value	Estimate (SE)	*p*-value
Intercept	2.865 (0.554)	0.00	2.675 (0.710)	0.00
Price	-0.635 (0.099)	0.00	-0.633 (0.099)	0.00
Unlabeled Healthy	0.317 (0.137)	0.02	0.317 (0.137)	0.02
Generic Label Healthy	0.953 (0.183)	<0.001	0.939 (0.183)	<0.001
Tailored Label Healthy	1.051 (0.212)	<0.001	1.106 (0.212)	<0.001
Control Label Healthy	0.220 (0.216)	0.31	0.188 (0.216)	0.38
Generic Label Unhealthy	-0.230 (0.189)	0.22	-0.242 (0.188)	0.20
Tailored Label Unhealthy	-0.810 (0.236)	<0.001	-0.758 (0.236)	<0.001
Control Label Unhealthy	0.032 (0.218)	0.88	-0.001 (0.217)	0.99
Wheat	0.200 (0.100)	0.05	0.197 (0.100)	0.05
Household Size			0.031 (0.023)	0.18
Male			0.277 (0.129)	0.03
Age			0.001 (0.004)	0.90
HH Income			-0.001 (0.004)	0.73
Primary Shopper			0.488 (0.187)	0.01
Education			-0.035 (0.029)	0.23
Log Likelihood	-1181.4		-1174.3	
Akaike Information Criterion	2384.8		2382.6	
Bayesian Information Criterion	2445.4		2476.3	
*n*	115		115	

Notes: Data are from the choice experiment and demographic survey.

**Table 3:** WTP estimates for healthy and unhealthy products by labeling condition, relative to an unhealthy product in the no label condition.

		95 Percent Confidence Interval	
	WTP	Low	High
WTP for Healthy Product			
No Label	0.501	0.079	0.922
Tailored Label	1.746	1.109	2.382
Control Label	0.296	-0.370	0.963
Generic Label	1.482	0.932	2.033
WTP for Unhealthy Product			
Tailored Label	-1.196	-1.921	-0.472
Control Label	-0.001	-0.674	0.671
Generic Label	-0.382	-0.963	0.198

Notes: WTP = willingness to pay.

## Appendix A

Healthy Food, Healthy Choice Food Labeling Research

Today, we are interested in asking you some questions about food choices. You will be presented with eight rounds of choices between two food products. After the eight choices, we will ask you to complete a short survey.

For each of the eight choices, you will view details about the two products, including a price. You will indicate which of the two products you would choose given the characteristics and prices of the products. You will also have the opportunity to indicate that you would choose to purchase neither product if faced with those two product options.

Though these choices are hypothetical, we ask that you think carefully about each decision, as though your choices were real. When you are faced with each choice in this survey, think about the other ways in which you might use the money that each item costs.

In each round, you will be presented a choice of two different breakfast cereals, A and B. Cereals A and B will be the same brand of cereal, but they will have different attributes. For each pair of cereals—and given the price and characteristics of each cereal—we would like you to indicate whether you would choose cereal A, cereal B, or whether you would choose not to purchase either cereal at the prices offered. The following are the attributes that will vary between the cereals:

Brand: All of the cereals presented are produced by Malt-o-Meal.

Price: The price is expressed in dollars per 40-ounce bag. It is the price you would pay for the bag of cereal you select.

Variety: Variety describes the grain and type of cereal that will be presented to you.

Healthy Choice Label:

A healthy choice label will accompany certain healthier cereals. This label will accurately reflect that the cereal is a significantly healthier option than the other cereal offered. Here is a picture of the label:

Nutrition Facts and Ingredients: The standard nutrition facts label and ingredient list that you would normally find on a package of cereal will be available to you. The information in these lists will accurately describe the nutritional attributes of the cereal.

Again, after you review each pair of items presented in the following scenarios, please indicate whether you would choose cereal A, cereal B, or whether you would choose not to purchase either cereal if you were required to pay the price associated with each cereal.

Please let the researcher know if you have any questions now or at any point during the research process.

**Choice Scenario A1.**

	**Alternative A**	**Alternative B**	**Alternative C**
Brand	Malt-O-Meal	Malt-O-Meal	Neither alternative A nor alternative B
Variety	Corn Flakes	Frosted Flakes	
Price ($/bag)	$5.99	$4.99	
Healthier Option			
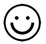
I would choose:	□	☐	□

## Appendix B

Appendix B: Example of the attribute combinations presented in eight choice sets in which unfrosted corn flakes were labeled as being a healthier choice, while unfrosted shredded wheat served as an unlabeled control. Note that the “Neither” option is not displayed.

**Choice Set**	**Type**	**Healthier?**	**Labeled**	**Price**	**Type**	**Healthier?**	**Labeled**	**Price**
1	Shredded wheat	Yes	No	$4.99	Frosted shredded wheat	No	NA	$4.99
2	Frosted corn flakes	No	NA	$5.99	Corn flakes	Yes	Yes	$5.99
3	Frosted shredded wheat	No	NA	$5.99	Shredded wheat	Yes	No	$4.99
4	Corn flakes	Yes	Yes	$5.99	Frosted corn flakes	No	NA	$4.99
5	Shredded wheat	Yes	No	$5.99	Frosted shredded wheat	No	NA	$4.99
6	Frosted corn flakes	No	NA	$5.99	Corn flakes	Yes	Yes	$4.99
7	Frosted shredded wheat	No	NA	$5.99	Shredded wheat	Yes	No	$5.99
8	Corn flakes	Yes	Yes	$4.99	Frosted corn flakes	No	NA	$4.99

## Appendix C

Appendix C: Proportions of healthier, less healthy, or “neither of these” choices made in each label condition in choice experiment

	**Healthier**	**Less healthy**	**Neither**
Unlabeled	0.450	0.380	0.170
Generic Label	0.604	0.330	0.066
Tailored Label	0.620	0.217	0.163
Control Label	0.431	0.387	0.182

Notes: All participants (*n* = 115) made decisions among “healthier”, “less healthy”, and “neither” in four choice sets that contained no labeled products, 49 participants made decisions among “healthier”, “less healthy”, and “neither” in four choice sets that contained healthier products labeled with the generic label; 35 participants made decisions among “healthier”, “less healthy”, and “neither” in four choice sets that contained healthier products labeled with the tailored label; and 31 participants made decisions among “healthier”, “less healthy”, and “neither” in four choice sets that contained healthier products labeled with the control label.
